# Map of Visual Activity in the Infant Brain Sheds Light on Neural Development

**DOI:** 10.1371/journal.pbio.1002261

**Published:** 2015-09-29

**Authors:** Janelle Weaver

**Affiliations:** Freelance Science Writer, Carbondale, Colorado, United States of America

## Abstract

A new study reveals that despite their limited visual acuity, 7-week-old babies have a surprisingly advanced visual cortex, including the ability to integrate visual and vestibular motion information. Read the accompanying Research Article.

Newborns enter the world equipped with the neural circuitry to see what’s around them, but their visual acuity is poor and most basic visual functions are immature. Many visual abilities, such as the perception of motion direction, begin to develop soon after birth. The perception of motion, color, and depth continue to mature as infants gain more experience with the world around them. However, our understanding of visual development in humans has been limited because there has been no direct functional evidence from awake infants showing how various visual cortical areas mature.

In a study published this week in *PLOS Biology*, Maria Concetta Morrone of the IRCCS Stella Maris Foundation and the University of Pisa and her collaborators localized for the first time cortical areas with high selectivity to visual stimuli in the first weeks of life. Using functional magnetic resonance imaging (fMRI) to record brain activity in awake seven-week-olds, they revealed an unexpected early maturation of the cortical system for motion processing. By providing the first functional maps of visual cortex in human infants, the study sheds new light on neural plasticity very early in life and could lead to the development of new rehabilitative strategies for neurodevelopmental disorders associated with impaired vision.

In the first fMRI experiment, Morrone and her team recorded brain activity in 12 awake, cooperative infants as they viewed random dot patterns that moved either randomly or coherently along radial, spiral, or contraction trajectories. When analyzing the data, the researchers looked for brain regions that showed greater responses to coherent motion compared to random motion. The network of areas activated in infants was very similar to that previously observed in adults. Both age groups showed an extensive motion-sensitive network of brain regions, including MT+ and V6—areas crucial for motion perception—as well as a vestibular brain region called PIVC/PIC (Fig 1).

**Fig 1:**
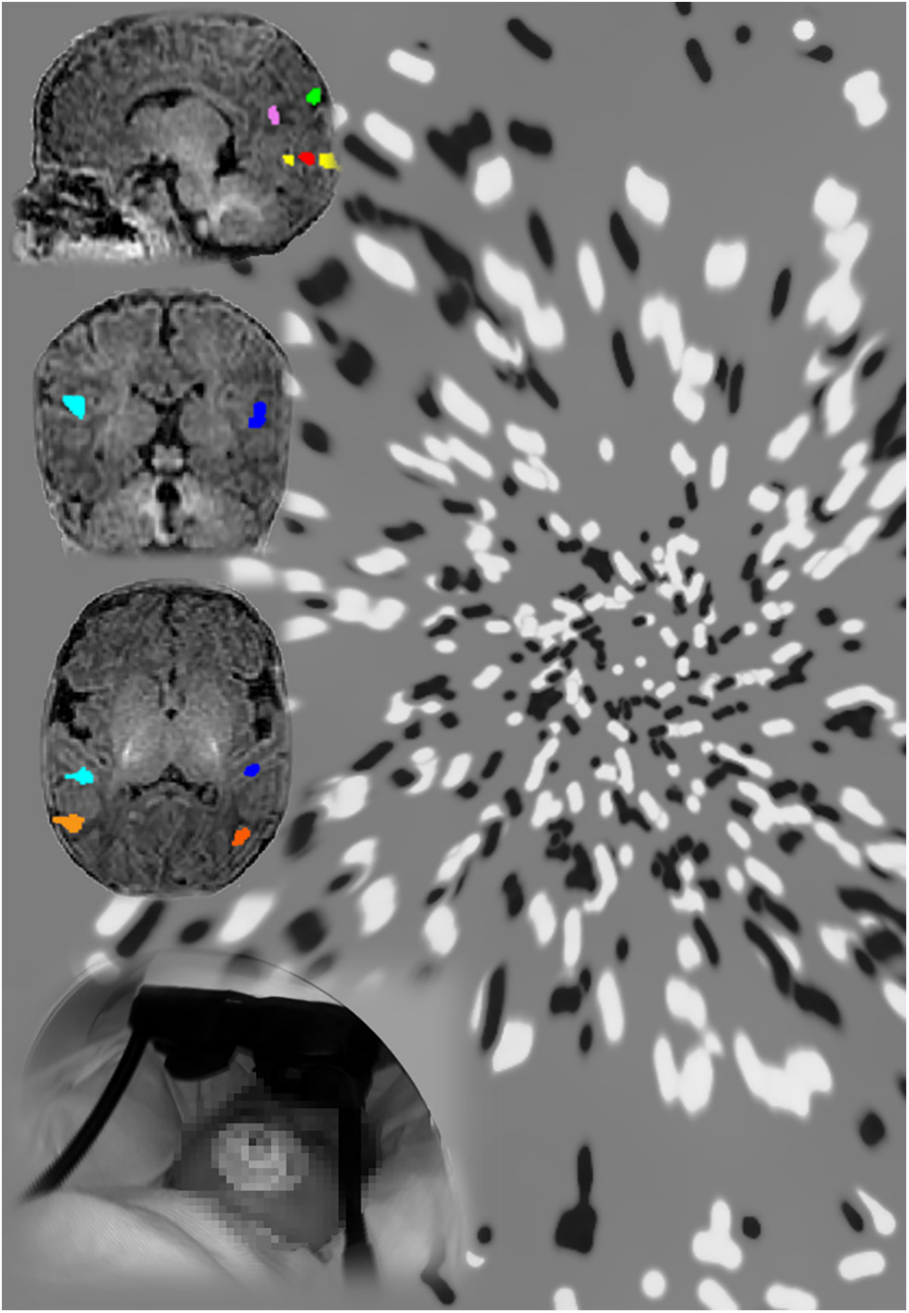
Very young infants in the scanner, while attentively observing visual motion, show activation in a coordinated network of visual areas that respond selectively to dots moving along a coherent trajectory, for example, the expansion or contraction produced by ego-motion. Image credit: Laura Biagi.

In nine of the infants, the researchers used fMRI to record resting-state brain activity during spontaneous sleep. When they analyzed activity patterns in the motion-sensitive regions identified in the first experiment, they found many similarities between infants and adults. For example, regions such as MT+ showed similar activity patterns in the left and right hemispheres of the brain in both age groups. But there were some notable differences. For example, the correlation between activity in V1 and MT+ was negative in adults but positive in infants, and PIVC/PIC also showed different activity patterns depending on the age of the subject.

Taken together, the findings demonstrate that the major cortical areas serving motion processing in adults are operative by seven weeks of age. Perhaps most surprising is the evidence that young infants may integrate visual motion with vestibular information to sense their own body position. On the other hand, the different patterns of activity between infants and adults suggest that this visual-vestibular connection is still developing during the first weeks of life, and other motion-related pathways also require more time to fully mature.

According to the authors, the findings may ultimately have important clinical implications. Vision is impaired in many neurodevelopmental disorders, such as autism and cerebral palsy. But the lack of knowledge about the precise location of different visual areas in the infant brain has made it difficult to predict the effects of brain damage in visual cortex. Studies that examine visual cortex at various stages of infant development could provide a much-needed brain atlas for this age group and shed new light on the mechanisms of neural plasticity. In the end, this information could help guide clinicians in the effort to select appropriate rehabilitation strategies in a timely manner to achieve optimal clinical outcomes.
